# Interaction of the solar wind with comets: a Rosetta perspective

**DOI:** 10.1098/rsta.2016.0256

**Published:** 2017-05-29

**Authors:** Karl-Heinz Glassmeier

**Affiliations:** Institut für Geophysik und extraterrestrische Physik, Technische Universität Braunschweig, Mendelssohnstraße 3, 38116 Braunschweig, Germany

**Keywords:** comets, solar wind interaction, space plasma, magnetic field draping, plasma waves

## Abstract

The Rosetta mission provides an unprecedented possibility to study the interaction of comets with the solar wind. As the spacecraft accompanies comet 67P/Churyumov–Gerasimenko from its very low-activity stage through its perihelion phase, the physics of mass loading is witnessed for various activity levels of the nucleus. While observations at other comets provided snapshots of the interaction region and its various plasma boundaries, Rosetta observations allow a detailed study of the temporal evolution of the innermost cometary magnetosphere. Owing to the short passage time of the solar wind through the interaction region, plasma instabilities such as ring--beam and non-gyrotropic instabilities are of less importance during the early life of the magnetosphere. Large-amplitude ultra-low-frequency (ULF) waves, the ‘singing’ of the comet, is probably due to a modified ion Weibel instability. This instability drives a cross-field current of implanted cometary ions unstable. The initial pick-up of these ions causes a major deflection of the solar wind protons. Proton deflection, cross-field current and the instability induce a threefold structure of the innermost interaction region with the characteristic Mach cone and Whistler wings as stationary interaction signatures as well as the ULF waves representing the dynamic aspect of the interaction.

This article is part of the themed issue ‘Cometary science after Rosetta’.

## 1. The classical interaction scenario

**Figure 1.**
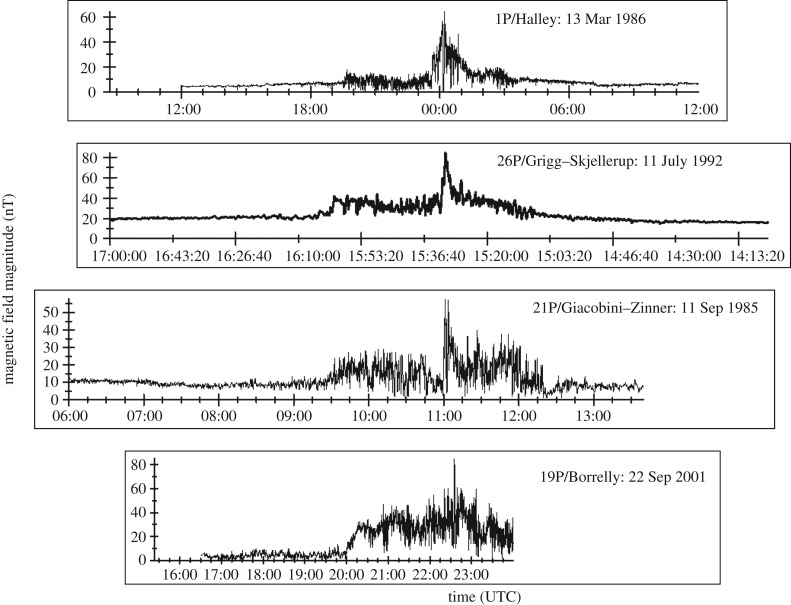
Magnetic field magnitude observations at comets 1P/Halley, 26P/Grigg–Skjellerup, 21P/Giacobini–Zinner and 19P/Borelly. Time scales are modified such that the closest approach graphically coincides at all four comets. The data shown are adapted from [10].

Cometary tails are one of the most fascinating features of any night sky. Without the existence of plasma and dust tails, we would not know about the existence of comets at all. A fully developed plasma tail always points radially away from the Sun, an observation that allowed Ludwig Biermann [1] to conjecture that cometary tails are due to the interaction of a cometary nucleus with a stream of particles, the solar wind. Hannes Alfvén [2] first suggested that the actual tail formation is due to draping of interplanetary magnetic field lines around the nucleus. In a further pioneering study, Biermann and co-workers [3] provided details of the comet--solar wind interaction by introducing the concept of mass, momentum and energy loading of the solar wind due to ionization of cometary neutrals released from the nucleus via sublimation. In the classical cometary case, mass loading is the dominant and most important effect. Implantation of cometary heavy ions into the solar wind requires momentum and energy transfer from the solar wind reservoir to these newborn particles. As the solar wind plasma is a collisionless medium, the implantation of the newborn ions requires a special physical process: coupling of the ions with solar wind protons and electrons via strong plasma waves and their electromagnetic field oscillations. Unstable phase space distributions generate these oscillations, first suggested by Wu & Davidson [4]. Magnetic field measurements of the International Cometary Explorer (ICE) during its flyby at comet 21P/Giacobini–Zinner [5] provided first observational evidence for strong plasma waves and turbulence in cometary environments. Later, observations at 1P/Halley, 26P/Grigg–Skjellerup and 19P/Borelly confirmed the Wu–Davidson conjecture [6–9]. Strong plasma turbulence is indeed the major characteristic of the interaction regime, as a comparison of measurements at the above-mentioned comets demonstrates (figure 1).

**Figure 2.**
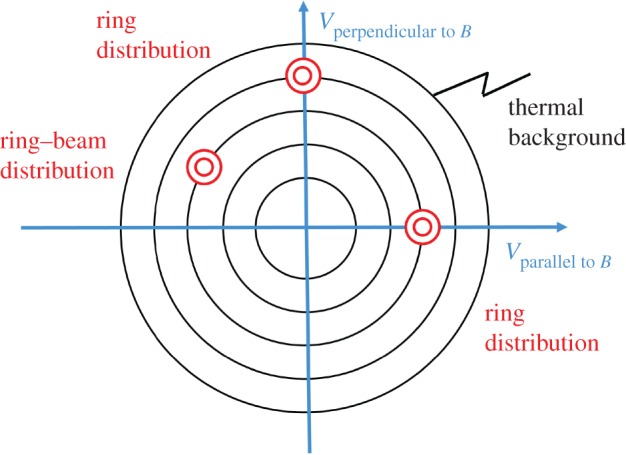
Schematic representation of a ring distribution, a beam distribution as well as a ring--beam distribution of heavy newborn cometary ions against the background of the thermal solar wind ion distribution. (Online version in colour.)

**Figure 3.**
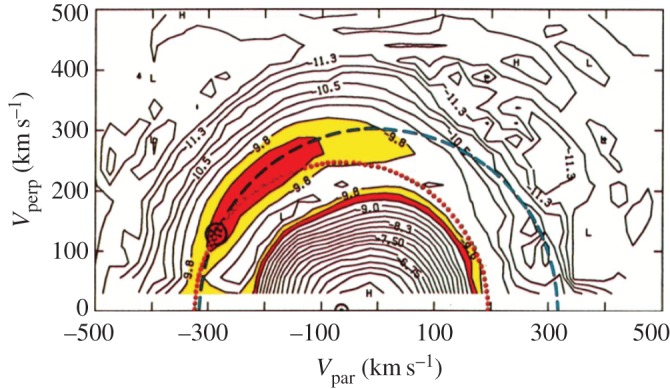
1P/Halley observation of a ring--beam pick-up proton distribution with scattering already forming a partial shell-like distribution (adapted from [14]).

The unstable phase space distributions caused by the newborn ions strongly depend on the angle between the solar wind flow ***u***_SW_ and the interplanetary magnetic field vector ***B***_IMF_. If the magnetic field is directed perpendicular to the solar wind flow, pick-up of the newborn ions is via the convectional electric field ***E***_CONV_ = −***u***_SW_ × ***B***_IMF_, which causes co-motion of the ions with the solar wind flow. In addition to this ***E* **× ***B*** drift, the ions of cometary origin gyrate around the local magnetic field. This initiates a ring distribution in velocity space (figure 2). If the magnetic field aligns with the solar wind flow, ***E* **× ***B*** pick-up is unimportant. In this case, the newborn ions represent a heavy-ion beam distribution in velocity space. For any other case, a ring--beam distribution forms (figure 2). In general these ring--beam distributions are unstable [11,12] and generate the plasma waves and turbulence necessary to facilitate the final incorporation of the implanted ions into the solar wind plasma. Scattering of pick-up ions by solar wind fluctuations and the self-generated plasma waves/turbulence eventually causes the build-up of shell-like ion distributions [13–15]. As an example, such a shell-like distribution is displayed for pick-up protons in figure 3.

This scattering is responsible for the final pick-up of the ions of cometary origin by the solar wind. Mass loading as anticipated by Biermann and co-workers [3] happens and causes deceleration of the flow as momentum and energy transfers from the solar wind to the cometary ions. Eventually, a bow shock forms at some position along the stagnation streamline, at that position where the mean molecular mass of the loaded plasma 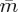 is increased by a factor *γ*^2^/(*γ*^2^ − 1) due to the pick-up ions. Here, *γ* = ( *f* + 2)/*f* is the polytropic index and *f* denotes the number of degrees of freedom. Once the increase of the mean molecular mass is greater than the critical value, a stationary solution for the mass-loaded flow no longer exists. A bow shock wave forms [3,16–18]. Shock formation is understood when considering momentum and energy conservation of mass loading. Consider a volume element with mass *m*_1_ and bulk velocity *u*_1_. Adding a mass 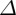 to this element and considering momentum conservation gives one a change in bulk velocity: 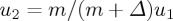. Now energy conservation requires 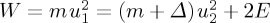, where *E* is an excess energy required to fulfil both momentum and energy conservation. This excess energy, 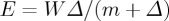, increases the internal energy of the flow. The internal energy reservoir depends on the number of degrees of freedom of the medium and thereby on the polytropic index *γ*. With increasing mass loading, this reservoir eventually fills up. The flow becomes shocked.

**Figure 4.**
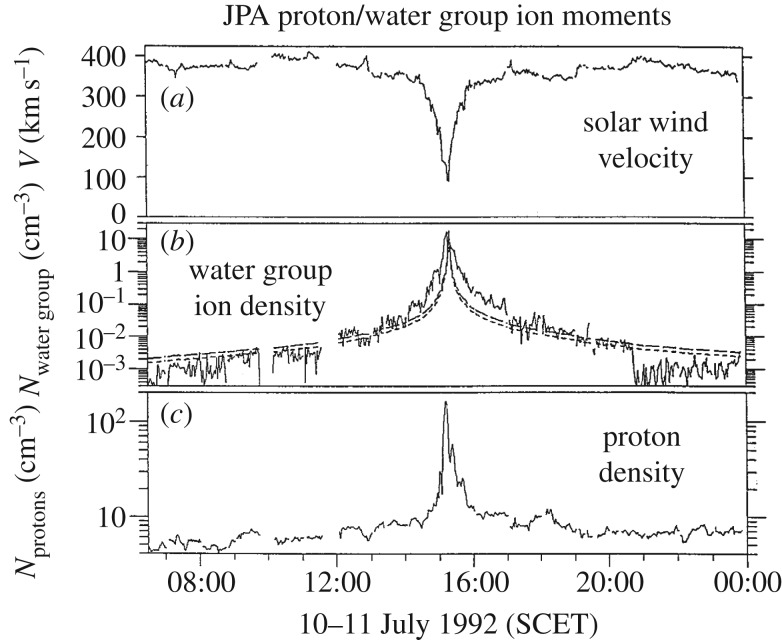
Solar wind flow velocity (*a*), water group ion density (*b*), and proton density (*c*) measured during the Giotto flyby at comet 26P/Grigg–Skjellerup (modified from [15]).

**Figure 5.**
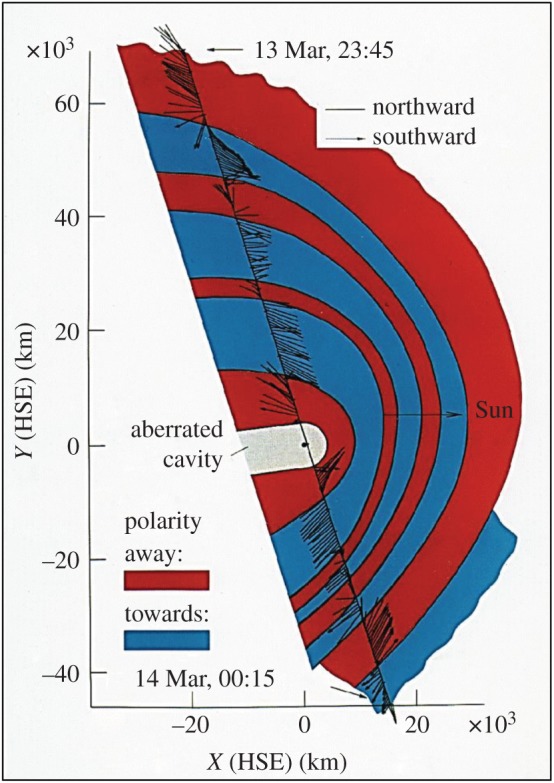
Draped magnetic field line regions around the nucleus of comet 1P/Halley. Projections of the magnetic field vectors (normalized to their magnitudes) on the *x–y* plane of the Halley-centred solar ecliptic (HSE) coordinate system are shown along the encounter trajectory of the Giotto spacecraft. The regions of opposite interplanetary magnetic field polarity are indicated by blue and red areas, where blue denotes field vectors pointing towards the Sun, red those with anti-sunward direction. (Data based on [19]; figure courtesy Fritz M. Neubauer.)

Flow diversion around the cometary object begins already within the shock, where thermalization and entropy production occur [17]. Behind the bow shock, the solar wind flow significantly decelerates and particle densities of both protons and cometary ions increase steadily (figure 4). Note that stagnation of the flow is not a necessary result of this deceleration and mass loading. However, magnetic field draping as predicted by Alfvén [2] has been observed by the magnetometer experiment on board Giotto [19] (figure 5). A series of magnetic field lines pointing in sunward and anti-sunward directions drapes around the nucleus. The series is most probably due to a succession of tangential discontinuities embedded in the solar wind and interacting with the comet. As mass loading decelerates the solar wind flow, the distance between the tangential discontinuities significantly reduces. In the 1P/Halley case, it is only about 5000–10 000 km.

Closer to the outgassing nucleus, the neutral gas density significantly increases. Direct interaction of the diverted and decelerated mass-loaded solar wind plasma occurs due to ion–neutral friction forces. A boundary, the magnetic cavity, exists where ion--neutral friction forces balance the magnetic forces of the plasma [20,21]. Such a magnetic cavity was first observed at comet 1P/Halley [22]. This short overview indicates the major features expected in the solar wind interaction region of an active comet, in the classical interaction scenario. For further details, reference is made to [23,24].

## 2. Rosetta observations

The Rosetta mission to comet 67P/Churyumov–Gerasimenko was launched in March 2004 [25]. It arrived at its target object in August 2014. Since this time, the spacecraft has been in the immediate cometary environment throughout 67P/Churyumov–Gerasimenko's journey around the Sun. One of the aims of the mission was to provide a detailed examination of the temporal evolution of the interaction region as described above. However, as Rosetta was always located in the inner coma of the comet, the temporal evolution of structures like the cometary bow shock could not be investigated.

Instead, a most detailed investigation of plasma physical processes in the inner coma is possible. Here, we concentrate on the physics of the interaction during the low- and intermediate-activity phase of the comet. We define as low- and intermediate-activity phase those phases of the mission where the activity causes an interaction region whose scale is significantly less than an ion gyroradius. At 1P/Halley the ratio of interaction scale (for example, the bow shock distance to the nucleus) to ion gyroradius was of the order of 100 (table 1). At 27P/Grigg–Skjellerup, it is still about six, while it is of the order of 0.005 for the Rosetta mission phases discussed here. We define the passage time of a solar wind plasma volume assuming a solar wind speed of 400 km s^−1^. The very different passage times for the various activity conditions are important for later discussions.

**Table 1.** Gyroradius, interaction scale and passage time for comets 1P/Halley, 27P/Grigg–Skjellerup and 67P/Churyumov–Gerasimenko together with their respective production rates [24,26].

comet	production rate (10^27 ^s^−1^)	cometary ion gyro- radius (km)	interaction scale (km)	passage time (s)
1P/Halley	690	10 000	1 115 000	2800
27P/G–S	7.5	4000	25 000	62
67P/C–G	0.2	37 000	1000	2.5

**Figure 6.**
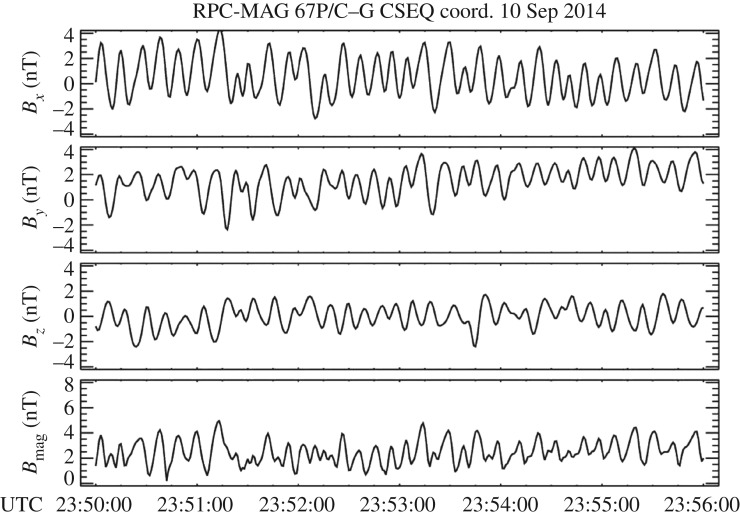
Magnetic field observations made by the RPC-MAG magnetometer experiment [10] on board the Rosetta spacecraft on 10 September 2015, 23.50–23.56 UTC.

When Rosetta arrived at 67P/Churyumov–Gerasimenko in August 2014, the most prominent features observed in magnetic field observations were quasi-harmonic oscillations of the magnetic field direction and magnitude (figure 6). Magnetic field measurements are displayed using a comet-centred solar equatorial (CSEQ) coordinate system, where the *x*-axis points from the comet to the Sun, the *z*-axis is the component of the Sun's north pole of date orthogonal to the *x*-axis, and the *y*-axis completes the right-hand system. The origin of the coordinate system is the centre of mass of the comet's nucleus. The amplitudes of these waves as measured by the magnetometer experiment on Rosetta [10] are rather large. The ratio of the perturbation amplitude of the magnetic field with respect to the ambient solar wind field strength, the δ*B*/*B* ratio, is significantly larger than 1. Spectral analysis reveals that the dominant frequencies of these oscillations, the ‘singing’ of the comet, range between 10 and 100 mHz [27,28]. A clear dependence of the wave frequency on magnetic field magnitude is not apparent. In this respect, the observed waves are very different from those observed earlier at comets 21P/Giacobini–Zinner, 1P/Halley, 26P/Grigg–Skjellerup and 19P/Borelly [5–9]. Any ring--beam instability during the low-activity phase of 67P/Churyumov–Gerasimenko is expected to generate waves of maximum amplitude 0.1 nT [29].

**Figure 7.**
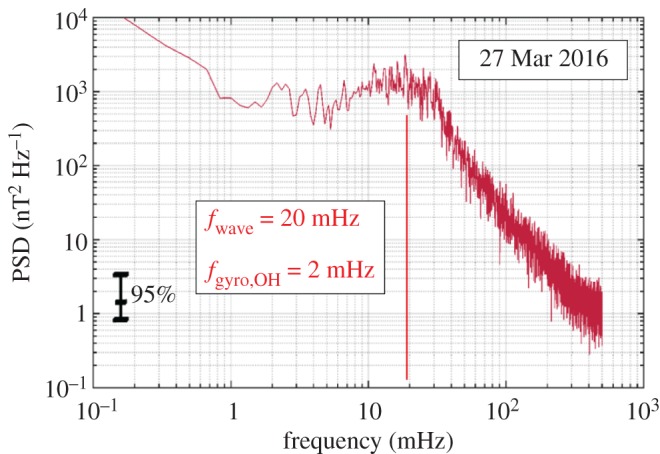
Sample spectrum of magnetic field oscillations made on 27 March 2016, after 67P/Churyumov–Gerasimenko passed perihelion. Displayed is the trace of the spectral density matrix. Wave frequency maximizes at about 20 mHz. The local water group ion gyrofrequency is 2 mHz.

The singing of the comet was observed until springtime 2015 or a distance of more than about 2.2 AU. The production rate of the comet at this distance was about 2 × 10^26^ s^−1^ [26]. Later, or closer to perihelion, the harmonic wave activity either disappeared or was buried in an environment of very large amplitudes (up to 270 nT), more erratic and chaotic magnetic field variations. However, at around February 2016, six months after perihelion passage, the singing reappeared, in that the larger-amplitude erratic variations are significantly reduced, the quasi-harmonic oscillations again becoming the dominant signature in the magnetic field observations. The production rate at the time of reappearance was again about 2 × 10^26^ s^−1^ [26]. Figure 7 displays a sample spectrum of the post-perihelion singing comet waves. The frequency of the oscillations is again significantly different from the local cometary ion gyrofrequency. These newly observed waves indicate a very different interaction scenario, as previously discussed for comets that are more active.

**Figure 8.**
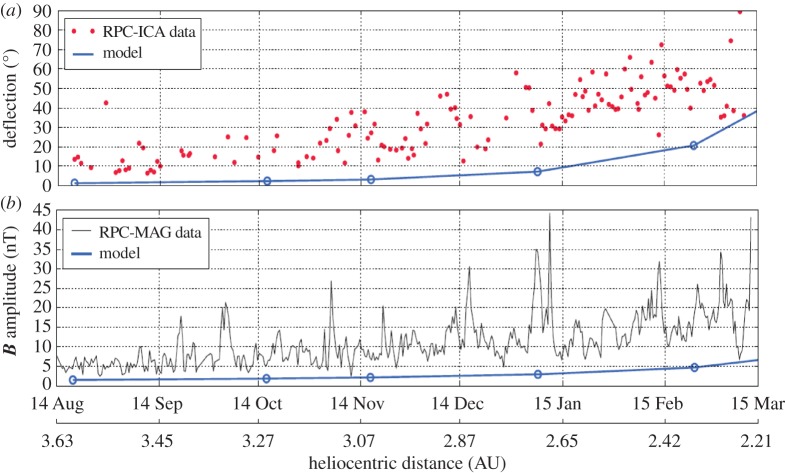
Temporal variation of the deflection angle (*a*) and the magnetic field magnitude (*b*) during part of the pre-perihelion phase of the Rosetta mission. The red dots give the daily median of the RPC-ICA measured deflection angle. The RPC-MAG magnetic field magnitude is averaged over 10 h. Blue lines display model results. (Figure courtesy Etienne Behar, based on [32].)

Another important observation is the clear deflection of the solar wind protons in the interaction region. Both the ion composition analyser RPC-ICA and the ion and electron sensor RPC-IES on board Rosetta [30,31] detected a significant deflection of the solar wind proton flow. Assuming that the solar wind flow is in the radial direction outside the comet interaction region, the deflection angle describes the deviation from this radial flow in the interaction region. Figure 8 indicates an increasing deflection with decreasing heliocentric distance, that is, with increasing cometary activity [32]. The deflection angle reaches a value of almost 90°. In parallel the magnetic field magnitude also increases, indicating at least some slowing down of the solar wind due to mass loading and associated magnetic field pile-up. RPC-IES sensor measurements also show a clear proton flow deflection during Rosetta's night-side excursion between 23 March and 10 April 2016. The deflection angle during this excursion varies with radial distance, reaching values of 80° close to the nucleus (K. Mandt, personal communication, 2016). This deflection is a direct consequence of the solar wind convectional electric field ***E***_CONV_ = −***u***_SW_ × ***B***_IMF_, accelerating newborn ions in the direction perpendicular to the local solar wind flow and interplanetary magnetic field. The average solar wind flow is in the radial direction from the Sun. Momentum balance requires deflection of the solar wind protons into the opposite direction, an effect already observed during the AMPTE barium cloud release and artificial comet generation experiment [33,34].

**Figure 9.**
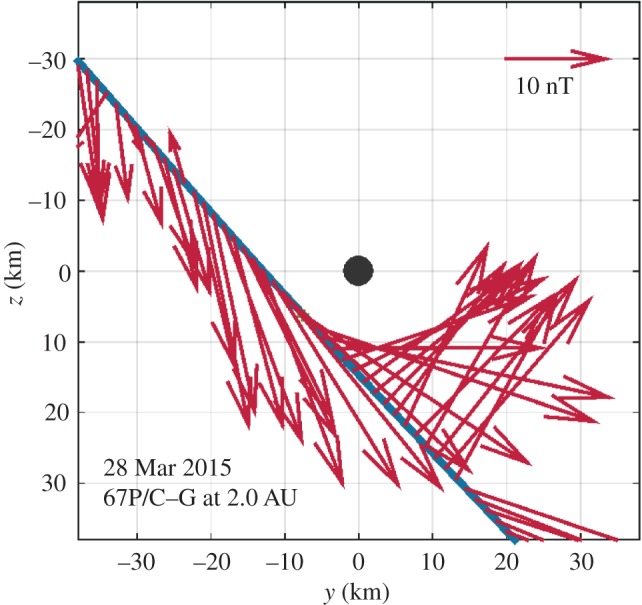
Magnetic field vectors projected onto the *y–z* plane of the CSEQ system during Rosetta's close flyby on 28 March 2016 (modified after [35]). Magnetic field measurements averaged over 60 s are used.

The deflection has also a profound impact on the draping of the magnetic field during this low-activity phase. Assuming that the magnetic field at a distance of about 2 AU is almost aligned with the azimuthal direction (that is, in the *y*-direction of the CSEQ system), the classical, mass loading-driven Alfvén-type draping causes the generation of a significant magnetic field component in the radial or *x*-direction. However, this is not observed during Rosetta's close flyby on 28 March 2016. During this flyby, Rosetta moved in an almost radial direction from distances of about 50 km to as close as about 15 km towards the nucleus (figure 9). At larger distances the *z*-component of the magnetic field dominates, pointing in the positive *z*-direction. Approaching closest approach, a significant *y*-component appears. After closest approach, the *z*-component dominates again, now pointing into the negative *z*-direction. During the interval shown in figure 9, the *x*-component is almost negligible. The flipping of the *z*-component from positive to negative direction is a clear signature of magnetic field draping. However, the dominant draping is not in the *x*--*y* plane as expected for a classical draping situation, but in the *y*--*z* plane. The draping observed in the present case is caused by deflection of the solar wind, not the classical mass loading [35].

**Figure 10:**
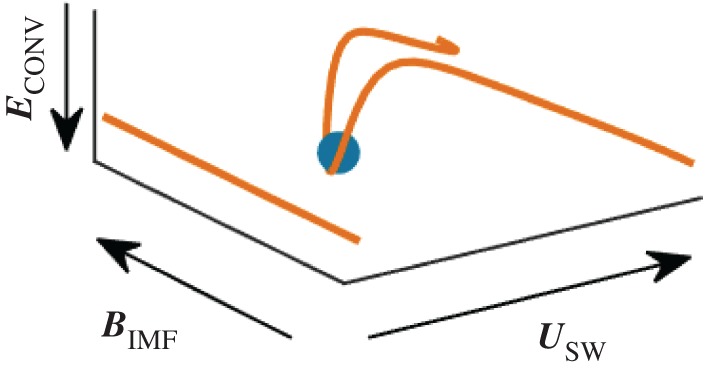
Schematic representation of magnetic field draping due to the pick-up induced proton flow deflection The ochre-coloured lines denote magnetic field lines, non-draped (left) and modified by the deflection (right). The blue circle denotes the nucleus. The solar wind flow direction ***u***_SW_, the direction of the interplanetary magnetic field ***B***_IMF_ as well as the associated convectional electric field ***E***_CONV_ are also indicated. (Adapted from [31].)

Figure 10 displays this situation schematically. Owing to the proton flow deflection, a magnetic field line originally aligned along the *y*-direction moves upwards into the *z*-direction near the nucleus. This causes the appearance of significant *z*-components, much as observed in the RPC-MAG measurements. As the field also drapes around the nucleus, a plasma tail structure emerges which is perpendicular to the comet--Sun line, not pointing in the radial direction as for very active comets [35,36].

## 3. Numerical simulations

**Figure 11.**
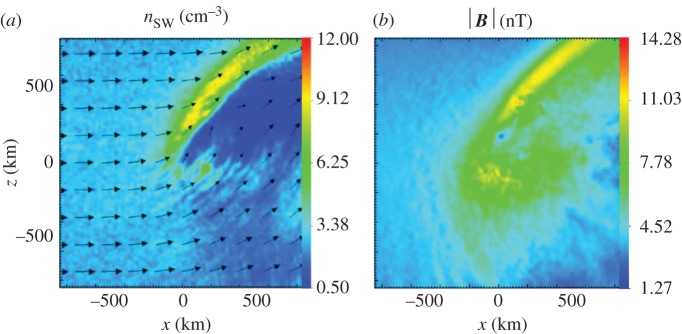
The plasma environment of 67P/Churyumov–Gerasimenko at 2.3 AU. (*a*) The solar wind density and velocity vector in the *x*–*z*-plane on the *y *= 0 cross section. (*b*) The corresponding magnitude of the magnetic field. (Adapted from [35].)

Numerical simulations allow a more detailed look into the physics of the deflection described [35,36]. Figure 11 displays the proton flow deflection discussed using a hybrid code numerical simulation. A standard set-up suitable for conditions at 67P/Churyumov–Gerasimenko is used. The interplanetary magnetic field is in the *x*–*y* plane at a Parker angle of 66°, while the solar wind flow aligns with the *x*-axis. A cometary production rate *Q* = 5 × 10^26^ s^−1^ is used, that is, this simulation describes the interaction for an intermediate-activity comet (for further details see [35]). For purposes of discussion of this and later simulation results, it is suitable to divide the interaction region into a +E-hemisphere and –E-hemisphere with respect to the *x*–*y* plane [35]. The +E-hemisphere is that region into which cometary ions are accelerated in the simulation. The cycloidal motion occurs in this region, characterized by negative *z*-coordinates. The –E-hemisphere is that region into which the protons deflect.

A clear increase of the proton density accompanies the deflection of the solar wind (figure 11). This increase is a consequence of mass density continuity. In the region of the density increase, the –E-hemisphere, the *B*_*z*_ component of the magnetic field is also significantly enhanced. In the magnetohydrodynamic (MHD) model of any plasma, a perturbation of the density is associated with a perturbation of the magnetic field magnitude if the perturbation is a fast-mode perturbation. Therefore, the observations point towards a fast-mode-type perturbation. The deflection is thus a fast-mode Mach cone structure in the –E-hemisphere [35]. For negative *z*-values, i.e. in the +E-hemisphere, there are indications for a decrease in density and magnetic field strength. Therefore, the Mach cone structure is asymmetric with respect to the *z* = 0 cross section. This constitutes a bilobate Mach cone, which is a cone with two lobes of different characteristics. The obstacle to the solar wind flow causing this Mach cone in the *x*–*z* plane is the momentum loading of the solar wind due to the ionization of cometary ions.

**Figure 12.**
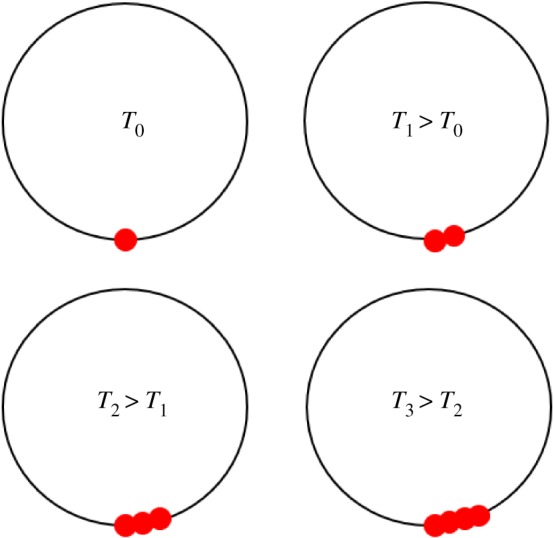
Schematic representation of phase space angle of newborn ions of cometary origin for the case of a very short passage or interaction time of a solar wind plasma volume and an intermediate-activity comet. (Online version in colour.)

The newborn ions not only constitute a mechanical obstacle to the solar wind, but they also act as an electric current source or obstacle. The interaction time of the solar wind and the cometary environment is extremely short, only a few seconds. Thus, the newborn ions constitute neither a ring--beam distribution nor a non-gyrotropic distribution [13,37]. The particular newborn ion distribution for the intermediate-activity situation discussed is better described as a delta-distribution with respect to the gyro phase angle or an extremely non-gyrotropic distribution (figure 12). For successive times *T*_3_ > *T*_2_ > *T*_1_ > *T*_0_, the new ions are incorporated into the solar wind plasma at phase angles which only slightly differ from each other. The newborn ions mainly constitute an electric current perpendicular to both the solar wind flow vector and the ambient magnetic field direction, tangential to the local gyro motion. The current direction coincides with the direction of the electric field ***E***_CONV_ = −***u***_SW_ × ***B***_IMF_, that is, the tangential current is a Pedersen current. This Pedersen current represents a disturbance to any local plasma currents already flowing in the cometary environment. Current closure requires the generation of a plasma wave. As the scale of the interaction region is smaller than a solar wind proton gyroradius, the wave excited cannot be an MHD wave. At these scales perturbations most likely propagate as Whistler waves. As the precise properties of the plasma are not known, a definite determination of the wave mode necessary to handle the perturbation of the plasma is not possible. We therefore preclude that Whistler-mode-type waves are excited.

A current disturbance in a plasma is a common phenomenon. For example, the relative motion of a natural satellite or spacecraft with respect to any magnetic field causes currents to flow, generating Alfvén or Whistler mode wings [38–40]. Thompson and co-workers [41] discuss the various wave modes excited by such a moving electric current source. Whistler mode waves preferentially propagate at an angle of about 19° with respect to the background magnetic field, which is almost along the magnetic field [42]. Wave phase propagation is superimposed by the solar wind propagation, resulting in downstream propagation and build-up of a Whistler wake structure around the electric current obstacle, in the plane spanned by the magnetic field and the flow. Such Whistler wing structures have already been observed in simulations of the solar wind interaction with asteroids [43].

**Figure 13.**
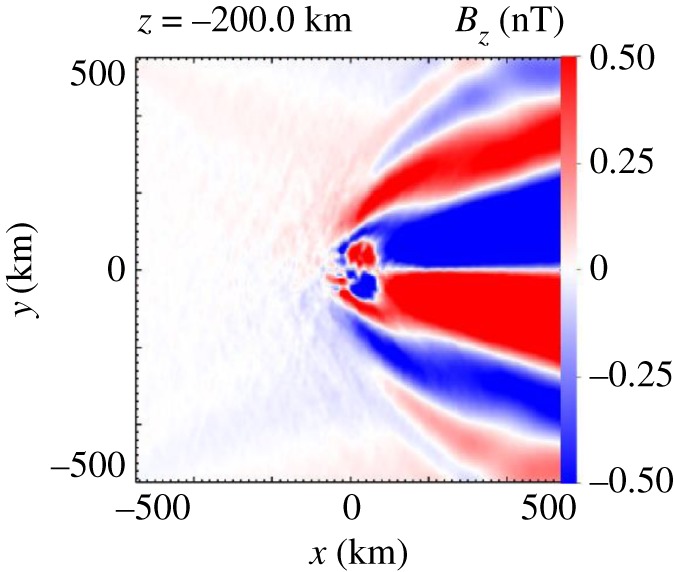
Distribution of the magnitude of the *B*_z_-component of the magnetic field in the +E-hemisphere (adapted from [35]).

The numerical simulations already discussed and used to demonstrate the generation of a bilobate Mach cone allow studies of the closure of the moving current disturbance and associated Whistler wing structure [44]. It should be noted that the current disturbance, the cross-field current generated by the implanted ions, is entirely located in the +E-hemisphere (figure 13). Therefore, current closure effects only occur in this hemisphere.

The simulation results displayed in figure 13 indicate the presence of a Whistler-wake-type structure with magnetic field variations of less than 0.5 nT. The simulation has been set up in such a way that the Whistler wake is clearly visible, without other effects hiding the wake structure [44]. Variations of the magnetic field as caused by the Whistler-type waves are difficult to discriminate from magnetic field variations from different sources because of their small amplitude. Therefore, unambiguous identification of the wake structure in the actual observations at 67P/Churyumov–Gerasimenko is not yet possible. This requires a more detailed analysis of joint observations of the various RPC sensors.

The Whistler-type waves expected to form the wake should also not be confused with the quasi-harmonic large-amplitude waves, the singing of the comet.

## 4. Singing of the comet and modified ion Weibel instability

The above discussion allows one to conclude that the interaction of a comet, in its low- to intermediate-activity phase, with the solar wind flow causes a bilobated fast-mode Mach cone structure in the plane, with the interplanetary magnetic field vector as its normal. In addition, a Whistler-type wake structure in the plane spanned by the solar wind flow vector and the magnetic field is evident from numerical simulations. A question that arises here is whether the shear flow associated with the proton flow deflection as well as the electric current flow constitutes stable situations in the cometary environment.

**Figure 14.**
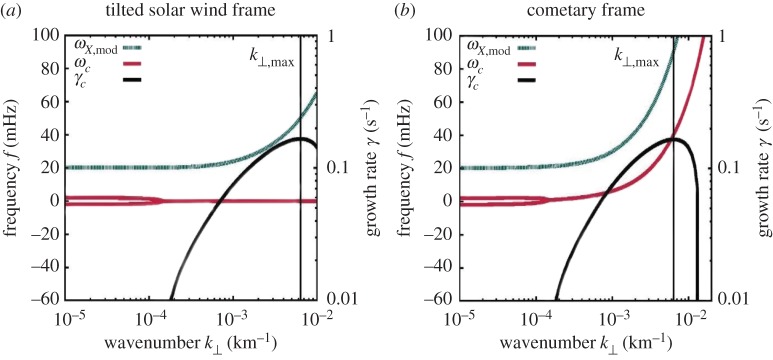
Dispersion diagram for wavevector direction perpendicular to both the ambient magnetic field and the solar wind flow. Dispersion properties in the tilted solar wind frame as well as the cometary frame are shown. The modified X-mode (green) is denoted *ω*_X,mod_. A purely growing cometary ion mode (red) is seen in the tilted solar wind frame (*a*) and transformed via Galileo transformation into a normal dispersion branch in the cometary frame (*b*). The growth rates of this ion mode are also given (black). The figure is modified from [45].

Meier *et al*. [45] offer an analytical treatment of the stability of the electric current flow. Using a cold plasma multi-fluid model, they provide an in-depth stability analysis. Their model allows study of wave excitation in a homogeneous three-component plasma (solar wind protons, electrons, and the implanted ions causing the electric current). A classical dispersion analysis, closely following earlier work on perpendicular electric current-driven instabilities [46–48], indicates that a modified ion Weibel instability is excited by the implanted ion current (figure 14). The unstable waves preferentially grow perpendicular to both the ambient magnetic field and this current. For reference, the modified X-mode is also displayed in figure 14. An unstable purely growing (in the solar wind frame) mode is generated due to the presence of the electric current density.

A note on the implanted ion and electron current density is appropriate here. In the CSEQ or cometary frame of reference, the newborn ions move perpendicular to the ambient magnetic field and the solar wind flow, while the newborn electrons move anti-parallel with the solar wind. As the solar wind velocity is much larger than the newborn ion velocity, the total resulting current density vector is almost anti-parallel to the solar wind flow. For the model plasma conditions used by Meier *et al*. [45], the tilt with respect to the solar wind flow direction is only 6°. Figure 14 displays the dispersion diagram for both the cometary frame as well as the tilted solar wind frame. The tilted frame has been introduced to ease the analytical computations [45]. This new frame corresponds to the solar wind frame of reference, but using a new *x*-axis, aligned with the electric current density direction.

The purely growing ion mode may be classified as a modified ion Weibel mode [45]. Weibel modes are electromagnetic waves self-generated in nearly homogeneous plasmas by ion or electron distributions that are anisotropic [46]. In the cometary case, the newborn ions and electrons cause the velocity anisotropy. It is of interest here to note that the Weibel instability is also considered as a process to explain the generation of any seed fields for dynamo action in the early Universe [49]. Ionization of neutrals emanating from cometary-type objects may have played a role in this.

The modified ion Weibel instability is a convective instability propagated by the solar wind [45]. For plasma parameters suitable for conditions at 67P/Churyumov–Gerasimenko during its intermediate-activity phase, the dispersion analysis provides maximum growth rates of the order of *γ* = 0.4 s^−1^ at a frequency of about 40 mHz and wavenumber *k* = 6.4 × 10^−3^ km^−1^ in the cometary frame of reference where the actual observations of the low-frequency waves are made [27,28]. The wavenumber at which maximum growth occurs corresponds to a wavelength of 980 km.

**Figure 15.**
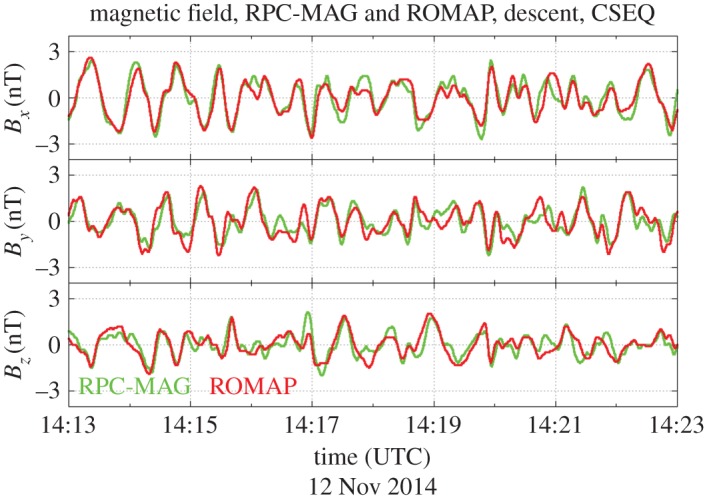
Magnetic field observations made on board the Rosetta spacecraft and its lander Philae during descent to the surface of the nucleus of comet 67P/Churyumov–Gerasimenko (adapted from [27]).

The observational conditions at 67P/Churyumov–Gerasimenko allow estimation of the wavelength, as two magnetometers, the magnetometer RPC-MAG on board the Rosetta spacecraft [10] and the ROMAP instrument on board the lander Philae [50], provide high-time-resolution observations of the magnetic field. Figure 15 provides an example of joint observations during the descent of Philae towards the surface of the nucleus. The magnetometers on board both spacecraft detected very similar low-frequency waves. The very good correlation between the two measurements is apparent. Both instruments recorded the same waves. As the distance of the two spacecraft changes during descent, a proper determination of the time shift between the signals can be done and a determination of the wavelength is possible [28]. The detailed analysis provides a value *λ* = (251 ± 31) km.

It should be noted that this value gives the wavelength projected onto the connection line between Rosetta and Philae. The actual wavelength may be greater than this value. The agreement with the theoretically determined value, *λ* = 980 km [45]*,* is already very reasonable and supports the idea that the singing of the comet is generated by a modified ion Weibel instability.

**Figure 16.**
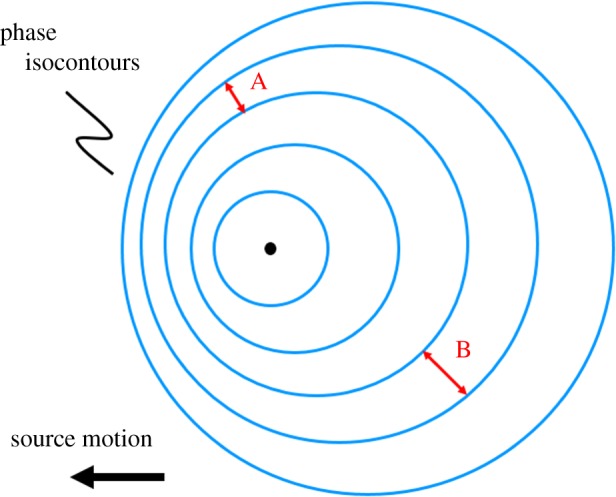
Schematic representation of the effect of source motion on the wave phase pattern. Phase isocontours (blue) pile up in the direction of source motion.

There is a further reason why any determination of the wavelength needs to be taken with care: the observationally determined wavelength depends strongly on the position at which the determination is done. Owing to the motion of the wave source with respect to the solar wind, the phase pattern of the wave field is rather complex (figure 16). In the direction of source motion, any detector intercepts wavefronts at a higher rate, the wavefronts pile up. In the opposite direction, the wavefront density diminishes. Determination of the phase difference between two phase isocontours at points A and B (figure 16) gives different values, depending on position. At point A, the distance between the phase isocontours is smaller than at point B, resulting in a smaller wavelength. Meier *et al*. [45] provide a more detailed discussion on this effect for the modified ion Weibel modes.

**Figure 17.**
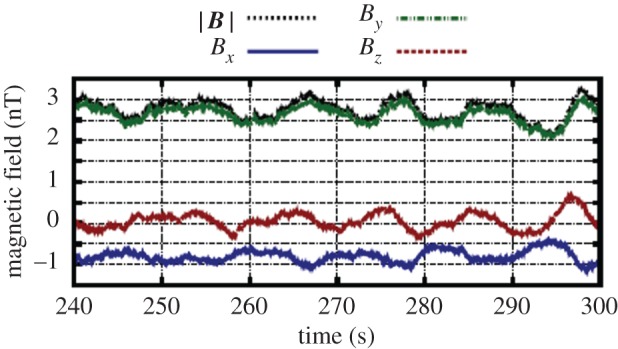
Simulated time series of the magnetic field fluctuations, taken from a point in the +E-hemisphere of the simulation box. The time interval was randomly selected. (Adapted from [44].)

Waves to be identified as modified ion Weibel modes have also been found in numerical simulations of the cometary situation discussed here [44]. These hybrid simulations, using the same code as in [35], reveal the existence of low-frequency waves with properties comparable to those observed at 67P/Churyumov–Gerasimenko as the singing of the comet. The waves (figure 17) are only detected in the +E-hemisphere, not in the –E-hemisphere. This points towards a generation mechanism related to the cross-field newborn ion current. Oscillations at a frequency of about 95 mHz (somewhat higher than actually observed, but comparable to the measured frequencies) are obviously generated in the interaction region. All magnetic field components oscillate with large amplitude. Similar oscillations occur in the electric field, the particle densities as well as the particle velocities [44].

**Figure 18.**
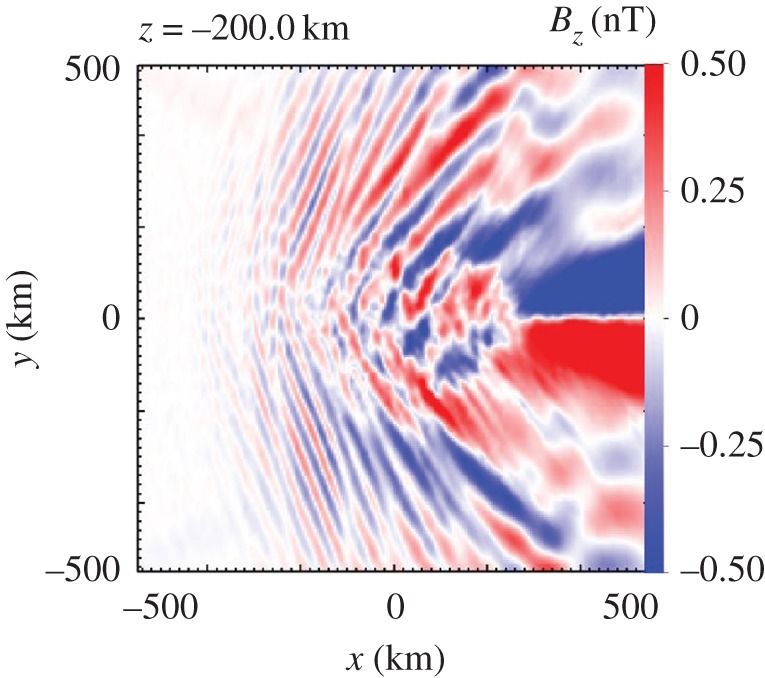
Spatial structure of the *z*-component of the magnetic field in the *x–y* plane of the +E-hemisphere at a distance of 200 km away from the nucleus of the numerical simulation (adapted from [44]).

The numerical simulations allow us to gain insight into the three-dimensional structure of the wave pattern. Superposition of the growing modes obviously results in a very interesting phase structure, a fan-like structure as displayed in figure 18. In addition to the night-side Whistler wake structure (figure 13), fan-like structures are generated by the unstable implanted ion current in front of the nucleus [44]. The distance between successive extremes of the *B*_*z*_ values is interpreted as the wavelength of the magnetic field oscillations. A value of 55 km is determined from the numerical simulation results [44]. The wavelength differs between various locations, larger values being observed further downstream.

Though this value is smaller than that identified using the magnetometer observations and that determined from the analytical computation, the fan-like structure should be associated with the ion Weibel modes [45]. A more systematic parameter study is under way to elucidate the plasma parameters controlling the wavelength and frequency.

## 5. Summary and conclusion

Mass loading controls the interaction of comets with the solar wind during the strong-activity phase of a comet. The scale of the interaction region, that is, the distance of the cometary bow shock to the nucleus, is large compared with any plasma scale such as the gyroradius of newborn ions. The passage or interaction time of a plasma parcel allows non-gyrotropic and ring--beam instabilities to create large-amplitude low-frequency waves at the cometary ion gyrofrequency in the cometary frame of reference [12,37]. The interaction time may also be large enough for strong turbulence to develop. Plasma waves and turbulence act as scattering agents to cause thermalization of the newborn ions and final pick-up by the solar wind. Ionization and subsequent mass loading is the major process to generate the cometary obstacle to the solar wind.

**Figure 19.**
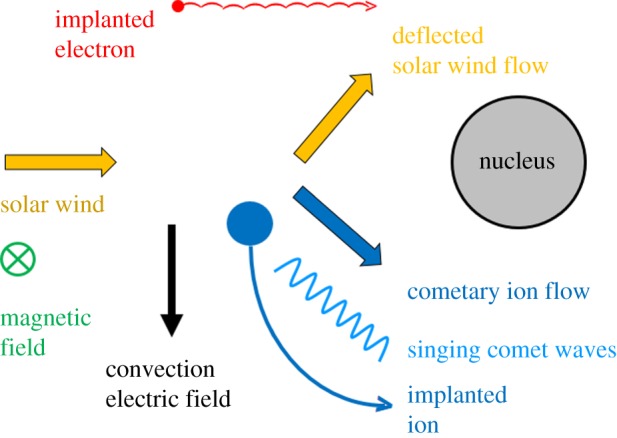
Schematic of the low- and intermediate-activity interaction of a comet with the solar wind.

During low and intermediate phases of activity, the more recent observations of Rosetta provide a new and different view on the interaction process and structure of the interaction region. Asymmetric deflection of the solar wind flow into the direction anti-parallel to the solar wind convectional electric field (already observed during the AMPTE barium release [33]) and large-amplitude, low-frequency magnetic field oscillation are the most striking features observed in the interaction region of the solar wind with comet 67P/Churyumov–Gerasimenko during its low- and intermediate-activity phase. The deflection results from momentum balance between the newborn ions, accelerated by the convectional electric field, and solar wind protons. Therefore, the newborn ions represent a mechanical disturbance to the flow. Ionization and momentum loading is a major process to generate an obstacle to the solar wind. As the passage time of a solar wind plasma volume across the outgassing comet is rather short compared with strong-activity situations, the implanted ions are essentially unmagnetized and do not cause any classical non-gyrotropic or ring--beam velocity space distributions, but they generate an electric current density perpendicular to the solar wind flow and magnetic field direction. This cross-field ion current constitutes an electric disturbance to the solar wind. Next to the mechanical obstacle, due to momentum loading, this electric current represents an electrodynamic obstacle to the solar wind (figure 19).

The mechanical obstacle causes a bilobate Mach cone structure in the wake of the comet. This Mach cone occurs in a plane that has the interplanetary magnetic field as its normal. In actual observations, this Mach cone has not yet been identified. However, it is apparent in numerical simulations of the interaction. The missing identification is due to unfavourable orbits of Rosetta around the cometary nucleus and the multitude of different phenomena and features observed in the interaction region.

The electrodynamics obstacle causes a Whistler-type wake structure in the plane spanned by the ambient magnetic field and the solar wind flow direction. This Whistler wake is easily identified in simulated data, but hardly detectable in the actual observations due to the small amplitude of the Whistler waves and unfavourable Rosetta orbits. However, the new type of low-frequency waves detected near the nucleus of 67P/Churyumov--Gerasimenko, the singing of the comet, should be interpreted as the result of the implanted ion-associated cross-field current being driven unstable. A modified ion Weibel instability is the most probable mechanism to drive the current unstable. Thus, a threefold interaction region picture emerges, with the Mach cone, the Whistler-type wake and the ion Weibel modes being the major signatures. Figure 19 tries to summarize the main features of this new type of interaction scenario.

**Figure 20.**
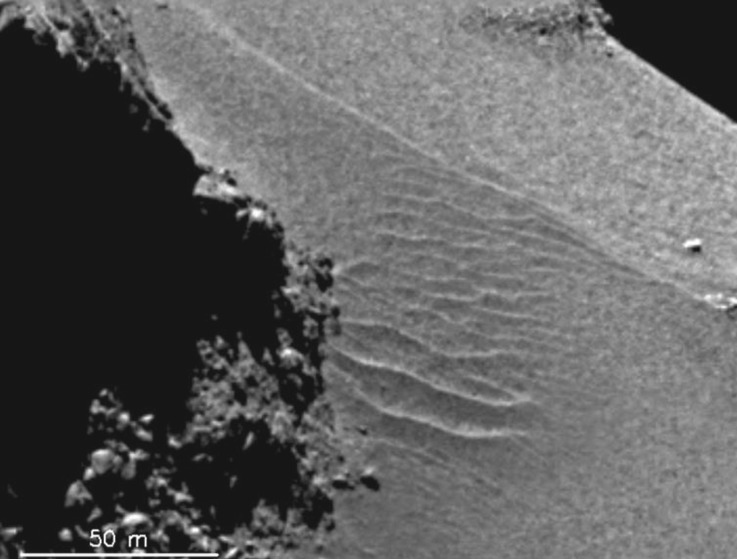
Aeolian ripples in the Hapi region at the surface of the nucleus of comet 67P/Churyumov–Gerasimenko (NAC_2014-09-18T00.33.01.377Z_ID10_1397549800_F22). The figure is adapted from [53].

There are further interesting and exciting observations made by the Rosetta Plasma Consortium [51] in the innermost interaction region of 67P/Churyumov–Gerasimenko. For example, multiple entries into a magnetic cavity have been observed in the interaction region [52]. However, their distance to the nucleus deviates significantly from those theoretically expected, which has caused an ongoing interesting scientific discussion. Other interesting observations concern electrically charged nanograins in the inner coma, indicating a possible connection between dust at the surface of the nucleus and the comet's plasma environment [53]. Aeolian ripples (figure 20) on the surface of the nucleus of 67P/Churyumov–Gerasimenko [54] may be another hint of a pronounced impact of the plasma environment on the nucleus morphology. The ripples observed exhibit a wavelength of about 10 m, which is comparable to the wavelength of ion acoustic waves in that environment [55]. Such ‘plasmaeolian’ structures would demonstrate the importance of a deeper understanding of the cometary plasma environment, if the conjectured relation can be confirmed.

Furthermore, the type of interaction described here may also be applicable to the interaction of Pluto with the solar wind. Whether the interaction is of the low to intermediate type discussed here, this is a relative characterization depending on the body's outgassing activity*.* The activity is classified as strong if the interaction scale, for example the bow shock distance, is large compared to the implanted ion gyroradius. Otherwise, the activity is low and intermediate. Furthermore, if the planetary body's scale is small compared to the implanted ion gyroradius, a cometary-type interaction as described here needs to be considered. At Pluto, this ratio is of the order of 1/500 [56].

Finally, one may speculate that the ion Weibel mode instabilities, used to interpret the singing of the comet phenomenon, may play a major role in magnetic field generation in the early Solar System. Ionization of neutral gases from outgassing planetesimals constitutes a source of velocity space anisotropy. This can drive magnetic field generation, much as conventional dynamo processes do [49].

## Data accessibility

Data presented here have been made available by the RPC team and are available through the PSA archive of ESA and the PDS archive of NASA. PSA is accessible via: ftp://psa.esac.esa.int/pub/mirror/INTERNATIONAL-ROSETTA-MISSION/.
